# Pterostilbene Attenuates Astrocytic Inflammation and Neuronal Oxidative Injury After Ischemia-Reperfusion by Inhibiting NF-κB Phosphorylation

**DOI:** 10.3389/fimmu.2019.02408

**Published:** 2019-10-17

**Authors:** Haixiao Liu, Xun Wu, Jianing Luo, Xiaogang Wang, Hao Guo, Dayun Feng, Lei Zhao, Hao Bai, Mingyang Song, Xunyuan Liu, Wei Guo, Xia Li, Liang Yue, Bodong Wang, Yan Qu

**Affiliations:** ^1^Department of Neurosurgery, Tangdu Hospital, The Fourth Military Medical University, Xi'an, China; ^2^Department of Neurosurgery, The 960th Hospital, Jinan, China; ^3^Department of Nursing, The 960th Hospital, Jinan, China

**Keywords:** cerebral ischemia-reperfusion injury, pterostilbene, nuclear factor-κB, astrocyte, inflammation, oxidative stress

## Abstract

Astrocyte-mediated inflammation and oxidative stress elicit cerebral ischemia-reperfusion (IR) injury after stroke. Nuclear factor (NF)-κB activates astrocytes and generates pro-inflammatory factors. The purpose of the present study is to elucidate the effect of pterostilbene (PTE, a natural stilbene) on astrocytic inflammation and neuronal oxidative injury following cerebral ischemia-reperfusion injury. A middle cerebral artery occlusion-reperfusion (MCAO/R) mouse model and HT22/U251 co-culture model subjected to oxygen-glucose deprivation and re-introduction (OGD/R) were employed, with or without PTE treatment. The data showed that PTE delivery immediately after reperfusion, at 1 h after occlusion, decreased infarct volume, brain edema, and neuronal apoptosis and improved long-term neurological function. PTE decreased oxidation (i.e., production of reactive oxygen species, malondialdehyde) and inflammatory mediators (tumor necrosis factor-α, interleukin-1β, and interleukin-6) and increased anti-oxidative enzyme activities (i.e., of superoxide dismutase, glutathione peroxidase), by inhibiting phosphorylation and nuclear translocation of NF-κB. In conclusion, PTE attenuated astrocyte-mediated inflammation and oxidative injury following IR via NF-κB inhibition. Overall, PTE is a promising neuroprotective agent.

## Introduction

Acute ischemic stroke (AIS) is the main cause of disability worldwide and is one of the leading causes of mortality (1). Treatments for AIS have made remarkable progress following the development of endovascular approaches and systemic thrombolysis (2). Further, there have been substantial advances in our knowledge of the pathophysiology of stroke (3). Moreover, we now know that inflammation associated with neuronal ischemia and reperfusion (IR) injury (IRI) plays a pathological role in stroke (3). Numerous neuroprotective drugs, however, have failed to show benefit in the treatment of IRI after AIS, making it imperative to search for new treatments (2).

Astrocytes, the most abundant glial cell type in the brain, provide metabolic and trophic support to neurons and modulate synaptic activity under normal physiological conditions (4). In stroke, however, studies have demonstrated that astrocytes contribute to the inflammatory response, which may aggravate the ischemic lesion, and form a glial scar, which may obstruct axonal regeneration and subsequently reduce the functional outcome (5). Accordingly, changes in astrocytic functions critically affect neuronal survival following stroke. Besides, cytokines such as interleukin (IL)-6 and IL-1, abundant in brain ischemia (6), are considered to induce astrogliosis (7). Activated astrocytes release multiple pro-inflammatory factors, including tumor necrosis factor-α (TNF-α) and IL-1β, which aggravate the injury after a stroke attack (8). Accumulating evidence indicates that astrocyte-mediated inflammation contributes to the secondary injury following cerebral IR (8, 9). Thus, targeting astrocytic inflammation after cerebral IR may prove to be a potential neuroprotective method following the recanalization of AIS.

Pterostilbene (3′, 5′-dimethoxy-resveratrol, PTE) is a natural stilbene derived from resveratrol that displays higher oral bioavailability and bioactivity but is far less abundant in natural sources (10). It exerts diverse pharmacological activities, including anti-inflammation, anti-oxidation, and anti-apoptosis (11, 12). In a previous study, we explored the benefits of PTE in attenuating inflammation and oxidative injury following subarachnoid hemorrhage and reducing mitochondrial oxidative stress after cerebral IR (11, 12). Other studies have also demonstrated the potent neuromodulatory effect of PTE on aging, Alzheimer's disease, and glioblastoma (13, 14). However, the role of PTE in modulating astrocyte-mediated inflammatory injury following cerebral IR has not been previously investigated.

Nuclear factor κ-light-chain-enhancer of activated B cells (NF-κB) is a transcription factor that plays a role in cell survival and inflammation (15). NF-κB is also involved in the pathological process of ischemic stroke, which can be activated by hypoxia, reactive oxygen species (ROS), and several inflammatory mediators (15). It also has the potential to modulate the production of multiple pro-inflammatory factors, including IL-6, IL-1β, and TNF-α in astrocytes (16, 17). Additionally, studies have reported that the selective inhibition of NF-κB could suppress astrocyte activation, subsequently down-regulating astrocyte-released chemokines and decreasing macrophage and T-cell infiltration and so reducing secondary inflammatory injury in central nervous system diseases (18, 19). Moreover, PTE has been reported to inhibit NF-κB signaling in the epidermis (20). Therefore, PTE may have an effect on astrocytes in the central nervous system through the regulation of NF-κB signaling.

The current study aims to clarify the protective effect of PTE on focal cerebral IRI following AIS and to explore the effect of PTE against astrocytic inflammation and related oxidative stress injury. Additionally, the effect of PTE on the phosphorylation and nuclear translocation of NF-κB in astrocytes is further discussed.

## Methods

### Animals

Male C57BL/6 mice, aged 8–12 weeks, weighing 20–25 g, purchased from the Laboratory Animal Center of the Fourth Military Medical University, China, were acclimated for 1 week in a temperature-constant room for 12 h light/dark cycles with free access to food and water. The animals were randomly allocated to the following groups: Sham + Vehicle, MCAO/R + Vehicle, MCAO/R + 5 mg/kg PTE, and MCAO/R + 10 mg/kg PTE. Experimental interventions and assays were performed by individuals who were blinded to this allocation. All experiments were conducted following the Guide for the Care and Use of Laboratory Animals issued by the U.S. National Institutes of Health and were approved by the Ethics Committee of the Fourth Military Medical University.

### Middle Cerebral Artery Occlusion-Reperfusion (MCAO/R) Model

MCAO/R surgery was performed as previously described (21). Briefly, the mice were anesthetized intraperitoneally with 2% pentobarbital (80 mg/kg body weight), followed by exposure of the right common carotid artery bifurcation, from which an uncoated 6-0 monofilament nylon suture (tip diameter 0.20 ± 0.01 mm) was inserted to obstruct the opening of the MCA. The distance from the bifurcation to the MCA was 10–12 mm, and the suture was removed after 1 h of occlusion. The animals were awake during the occlusion. The sham group underwent the same procedures except that the suture was not inserted. During the surgery, the rectal temperature of the mouse was maintained at 36.5 ± 0.5°C. The neurological score was assessed immediately after surgery when the animal had totally recovered from the anesthesia. To ensure homogeneity among groups, mice with a neurological score of >13 or <7 before drug administration were exempted from the following experiments.

### Drug Administration

According to the group allocations mentioned above, mice were administered either vehicle or PTE, intraperitoneally, at 1 h after surgery and either sacrificed after 24 h for histological and molecular biologic assays or treated with vehicle or PTE daily for behavioral testing. PTE (purity ≥98.0%; Sigma-Aldrich, St. Louis, MO, USA) prepared in dimethyl sulfoxide (DMSO) was diluted with normal saline in advance. In vehicle groups, mice received normal saline with DMSO.

### Cerebral Infarct Volume

Cerebral infarct volume was measured by using 2, 3, 5-triphenyltetrazolium chloride (TTC) staining as previously described (8, 22). Briefly, brains were rapidly removed, frozen, and sliced into 1-mm-thick coronal sections, dipped in 2% TTC (37°C, 30 min), and immersed in 4% paraformaldehyde. Ischemic (white) and normal (red) areas were analyzed with Image-Pro Plus 6.0, and the infarction proportion was calculated as follows: (Red area on left—Red area on right)/Red area on left (23).

### Brain Water Content

Brain water content was measured by using the standard wet-dry method with a modification (24). Right cerebrums were separated 24 h after reperfusion and weighed (wet weight), then dehydrated in an oven (105°C, 72 h). The dry samples were then weighed, and the brain water content was calculated as follows: (wet weight -dry weight)/wet weight.

### Neurological Score

The 18-point Garcia grading score was used to measure neurological function daily, as previously described (25), by an observer who was unaware of the group allocation. In brief, the scoring contained motor and sensory tests that highlight absent and abnormal movements. Higher scores indicated better neurological function. Scores of 1–6 indicates severe injury; 7–12, moderate injury; 13–18 mild injury.

### Cell Culture, PTE Treatment

Hippocampal neuronal HT22 and astrocytoma U251 cells were cultured in Dulbecco's modified eagle medium (DMEM) with 10% fetal bovine serum (FBS) (37°C, 5% CO_2_), which was replaced by FBS-free DMEM 24 h before the experiment. Culture media were changed every 2 days. Cell culture reagents were obtained from Gibco (Grand Island, NY, USA). PTE dissolved with DMSO was diluted in DMEM in advance (DMSO ≤ 0.1%). Before OGD/R, U251 cells were treated with PTE (2.5 or 5 μM) or vehicle for 4 h.

### HT22/U251 Co-culture and OGD/R Model

The HT22/U251 co-culture was based on a transwell co-culture system described previously (26, 27). U251 cells at a density of 2 × 10^5^ on transwell inserts (pore size 0.4 μm, polylysine-coated polycarbonate membrane; Corning, NY, USA) were placed above HT22 cells in a 24-well plate. The intervention was only administered to U251 cells. The under-layer HT22 cells were harvested 24 h after co-culture.

The OGD/R model was performed as previously reported (27). Briefly, the media were changed to glucose-free DMEM, and the cells were transferred to a hypoxic chamber (95% N_2_, 5% CO_2_, 37°C) for 2 h, followed by quick reintroduction of oxygen and glucose. Cells were harvested at 24 h after OGD/R.

### CCK-8 Assay

Cellular viability was measured with a CCK-8 assay kit (Dojindo Molecular Technologies, Kumamoto, Japan) according to the protocol described in its technical manual. Briefly, the cells in the 24-well plates were incubated with 10% CCK-8 solution at 37°C for 3 h. Next, 100 μL of supernatant per well was transferred into a 96-well plate for optical density (OD) detection with a microplate reader (Spectra Max M5, Molecular Device, USA) at 450 nm.

### Histology and Cytology

Tissue preparation was performed as described previously (12). Animals were anesthetized and perfused via the left ventricle with 30 mL of ice-cold 0.1 mol/L phosphate buffered solution (PBS; pH = 7.4) and continuous 20 ml 4% paraformaldehyde (dissolved in PBS). Brains were post-fixed in 4% paraformaldehyde and sequentially dehydrated in 20–30% sucrose solutions at 4°C for 24 h. Samples were sliced into 30-μm-thick coronal sections using a freezing microtome (CM1950, Leica) and mounted on polylysine-coated glass slides.

Cells cultured on carry sheet glasses in 24-well plates were washed with PBS, fixed with 4% paraformaldehyde for 30 min, and stained according to experimental design.

### ROS Staining, MDA, SOD, GSH-Px Assay, and ELISA Assay

The ROS fluorescent probes dihydroethidium (DHE) and dichlorofluorescein (DCF) were applied as previously reported (12, 28). In brief, fresh sections were incubated with 10 μmol/L DHE for 30 min, and cells grown on glass slides in 24-well plates were detected 24 h after treatment, incubated with 10 μM DCF at 37°C for 30 min in a dark incubator, and observed under a confocal microscope (FV1000, Olympus, Tokyo, Japan). ROS-positive cells were counted using Image-Pro Plus 6.0 by an observer blinded to group allocation.

Brain tissue collected from the peri-infarct area was lysed in lysis buffer (Beyotime Institute of Biotechnology, China) on ice for 30 min and centrifuged at 12,000 rpm at 4°C for 15 min to obtain the supernatant. The levels of MDA, SOD, and GSH-Px activity were then assessed using the appropriate kits, according to the manufacturer's instructions (Institute of Jiancheng Bioengineering, Nanjing, Jiangsu, China).

The levels of TNF-α, IL-6, and IL-1β in the culture media were measured by using ELISA kits (Nanjing KeyGEN Biotech Co. Ltd., Nanjing, China) according to the manufacturer's instructions.

### Immunofluorescence and TUNEL Staining

Immunofluorescence staining on brain and cell slices was performed as previously described (8, 12). Slices were treated with 0.3% Triton X-100 for 30 min and 10% donkey serum for 2 h, followed by incubation with primary antibodies at 4°C overnight. The primary antibodies were diluted with PBS as follows: rabbit anti-NeuN (1:300, Abcam), goat anti-GFAP (1:1000, Abcam), rabbit anti-p-p65 (S536) (1:100, CST), and rabbit anti-p65 (1:400, CST). The secondary antibodies (1:200, Abcam) were as follows: donkey anti-rabbit IgG (Alexa Fluor 594), donkey anti-goat IgG (Alexa Fluor 594), donkey anti-mouse IgG (Alexa Fluor 594), and donkey anti-rabbit IgG (Alexa Fluor 488). DAPI was obtained from Sigma (Saint Louis, MO, USA). Negative controls without primary antibody were performed for all samples.

TUNEL assay was used according to the manufacturer's protocol (Roche, Mannheim, Germany). Double-staining of TUNEL/NeuN was performed as previously described (22). In brief, the sections were initially stained with NeuN (1:1000, Abcam) and subsequently stained with TUNEL.

The sections were imaged using confocal microscopy. Five representative visual fields were randomly chosen to be analyzed, from the penumbra (**Figures 7C,D**) in each slice or from each dish. The fluorescent images were analyzed using Image-Pro Plus 6.0 or ImageJ by an observer who was blinded to grouping.

### Western Blot Analysis

Mice were perfused with ice-cold PBS, 24 h after MCAO/R, and brain tissue in the peri-infarct area was quickly separated on ice. The peri-infarct area was drawn using previously published methods (29). Cells were harvested at 24 h after OGD/R using a cell scraper. Samples were lysed in 10 μl/mg lysis buffers containing protease and phosphatase inhibitors (Roche, Mannheim, Germany). Nuclear and cytoplasmic proteins were extracted using nuclear and cytoplasmic extraction kits (Thermo Scientific Inc., USA). Bicinchoninic acid assay quantification and western blot analysis were performed as previously reported (12, 28). The amount of protein loaded was 30 μg per lane. The following antibodies were employed: anti-TNF-α (1:1000, Abcam), anti-IL-6 (1:1000, Abcam), anti-IL-1β (1:1000, Abcam), anti-GFAP (1:1000, Abcam), anti-p-p65 (S536) (1:1000, CST), anti-p65 (1:1000, CST), anti-ACTB (1:3000, Bioworld), anti-histone H3 (1:3000, CST), and secondary HRP-labeled antibodies (Bioworld Technology Inc., MN, USA). Data were scanned with an imaging system (Bio-Rad, Hercules, CA, USA) and analyzed with ImageJ software (version 1.46).

### Statistical Analysis

GraphPad Prism 5.0 (GraphPad Software Inc., USA) was used to analyze data. Results are expressed as the mean ± standard deviation (SD) unless otherwise noted. Comparisons among groups were assessed by one-way or two-way analysis of variance (ANOVA) and log-rank test, depending on the experimental design. Multiple comparisons were performed using *post-hoc* Tukey's honestly significant difference (HSD) test for significant ANOVA tests. Differences were considered statistically significant when *p* < 0.05.

## Results

The neurological scores of all mice modeling middle cerebral artery (MCA) occlusion-reperfusion (MCAO/R) was obtained. A total of 217 animals were considered to be at the same level of damage. Others (no more than 30%, whose neurological score was >13 or <7) were excluded from the study. In all groups, PTE or vehicle was delivered immediately after reperfusion, namely, 1 h after the stroke attack.

### Effect of PTE on Morphology and Function in MCAO/R Mice

**Figure 1:**
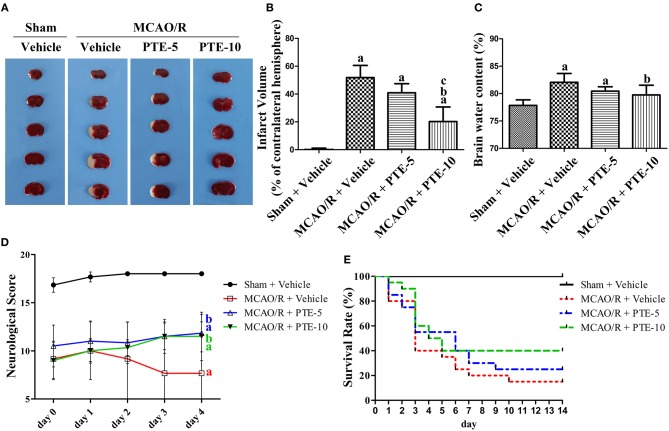
Cerebral infarct volume, brain edema, and neurological outcomes in MCAO/R mice with or without PTE administration. **(A)** Cerebral infarct volume was assessed by 2, 3, 5-triphenyltetrazolium chloride (TTC) staining at 24 h after MCAO/R or sham operation, and **(B)** the infarct volume ratio was calculated for each group. **(C)** The brain water content of the infarcted hemisphere (without epencephalon) was analyzed to evaluate cerebral edema. Values are expressed as mean ± standard deviation (*n* = 8). ^a^*p* < 0.05, compared with Sham + Vehicle. ^b^*p* < 0.05, compared with MCAO/R + Vehicle. ^c^*p* < 0.05, compared with MCAO/R + PTE-5. Significance was determined using a one-way analysis of variance. **(D)** Neurological function was assessed using the Garcia 18-point grading system at 2 h and 1, 2, 3, and 4 days **(D)** after MCAO/R or sham operation; the mice that died within 4 d after the operation were excluded from the analysis. Values are expressed as mean ± standard deviation (*n* = 8). ^a^*p* < 0.01, compared with Sham + Vehicle at day 3 and day 4. ^b^*p* < 0.01, compared with MCAO/R + Vehicle at day 3 and day 4. Significance was determined using a two-way analysis of variance. **(E)** The 14-day survival rate was analyzed for each group. PTE (at a dose of 5 mg/kg and 10 mg/kg for the PTE-5 and PTE-10 groups, respectively) and the same volume of vehicle were administrated every day after surgery. Values are expressed as the survival percentage (*n* = 20). Significance was determined using a log-rank test. MCAO/R, middle cerebral artery occlusion and reperfusion; PTE-5/10, pterostilbene 5 or 10 mg/kg.

In a previous study, we identified that a PTE injection at 10 mg/kg per day for five days had no effect on neurological scores and brain water content in normal mouse brains (11). 2, 3, 5-triphenyltetrazolium chloride (TTC) staining was used to measure the infarct volume of MCAO/R mice (Figure 1). The infarct volume and brain water content in the MCAO/R group (51.85 ± 8.723, 82.07 ± 1.611) were significantly higher than in the Sham group (0.1250 ± 0.9878, 77.85 ± 1.013). PTE (5 or 10 mg/kg), however, reduced the infarct volume (40.90 ± 6.509, 20.23 ± 10.44) and brain water content (80.45 ± 0.7868, 79.75 ± 1.7812) 24 h after reperfusion (Figures 1B,C, *p* < 0.05).

A total of six out of 30 animals were excluded from the neurological scoring assay since two mice in the Vehicle group died at day 1, and another two mice in the Vehicle group and two mice in the PTE-10 group died at day 3. The neurological score of the MCAO/R group decreased from day 0 to day 4 compared with the Sham group. PTE (both 5 and 10 mg/kg) administration significantly improved neurological scores compared with MCAO/R + Vehicle group at day 3 and day 4 (Figure 1D, *p* < 0.01). Moreover, the 2-week survival rate in the MCAO/R + Vehicle, MCAO/R + PTE-5, and MCAO/R + PTE-10 groups, were 3, 5, and 8 out of 20, respectively (Figure 1E). Although the survival rate doubled in the PTE-10 group compared with the MCAO/R + Vehicle group, this difference was not statistically significant (*p* > 0.05).

### Effect of PTE on Neuronal Apoptosis in the Peri-infarct Area of MCAO/R Mice

**Figure 2:**
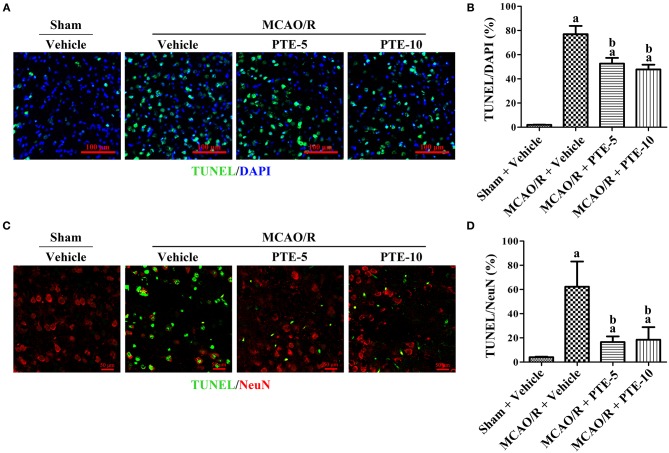
Neural apoptosis in the peri-infarct area at 24 h after MCAO/R with or without PTE administration. **(A)** Immunofluorescence double-staining of TUNEL (green) and DAPI (blue) was performed on frozen sections fixed at 24 h after MCAO/R or sham surgery. Scale bars = 100 μm. **(B)** The percentage of TUNEL-positive cells in the peri-infarct area was counted under fluorescence microscopy as an average of five visual fields. **(C)** The neuronal apoptotic rate was assessed using immunofluorescence double-staining of apoptotic cells (TUNEL, green) and neurons (NeuN, red) under the same conditions. Scale bars = 50 μm. **(D)** The percentage of TUNEL-positive neurons in the peri-infarct area was counted under fluorescence microscopy as an average of five random visual fields. Values are expressed as mean ± standard deviation (*n* = 8). ^a^*p* < 0.01, compared with Sham + Vehicle. ^b^*p* < 0.01, compared with MCAO/R + Vehicle. Significance was determined using a one-way analysis of variance. MCAO/R, middle cerebral artery occlusion and reperfusion; DAPI, 4′,6-diamidino-2-phenylindole; PTE-5/10, pterostilbene 5 or 10 mg/kg; TUNEL, terminal deoxynucleotidyl transferase dUTP nick-end labeling.

Terminal deoxynucleotidyl transferase dUTP nick-end labeling (TUNEL)-staining is a marker of apoptotic nuclei (12, 22, 30). We employed double-staining of TUNEL and 4′,6-diamidino-2-phenylindole (DAPI, which stains all nuclei) to assess the total apoptotic rate (Figure 2A) in the peri-infarct area of MCAO/R mice with or without PTE administration. Further, double-staining of TUNEL and NeuN (a neuronal nuclear antigen marker) was used to evaluate the neuronal apoptotic rate (Figure 2C). The total and neuronal apoptotic rates increased after MCAO/R. In comparison with that in MCAO group the total and neuronal apoptotic rates were lower in the MCAO/R + PTE groups (Figures 2B,D, *p* < 0.01).

### Effect of PTE on Neural Oxidative Stress and Inflammation in the Peri-infarct Area of MCAO/R Mice

**Figure 3:**
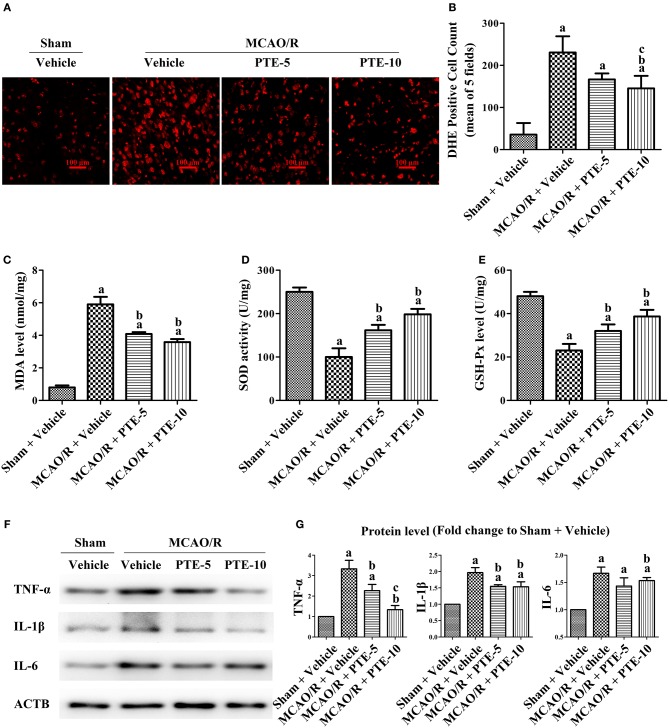
Oxidative stress and inflammatory levels in MCAO/R mice with or without PTE administration. **(A)** The cerebral oxidative stress level in the peri-infarct area was assessed using DHE-staining (red) at 24 h after MCAO/R or sham surgery. Scale bars = 100 μm. **(B)** DHE-positive cells in the peri-infarct area were counted by an observer blinded to the group assignments and analyzed as the average of five random visual fields. **(C–E)** The levels of MDA, SOD, and GSH-Px in the infarcted hemisphere were assessed using relevant test kits at 24 h after MCAO/R or sham. **(F,G)** The levels of pro-inflammatory factors TNF-α, IL-6, and IL-1β in the peri-infarct area were analyzed using western blotting at 24 h after surgery. ACTB was used as loading control, and the protein level was normalized to the Sham + Vehicle group. Values are expressed as mean ± standard deviation (*n* = 6). ^a^*p* < 0.05, compared with Sham + Vehicle. ^b^*p* < 0.05, compared with MCAO/R + Vehicle. ^c^*p* < 0.05, compared with MCAO/R + PTE-5. Significance was determined using a one-way analysis of variance. ACTB, β-actin; MCAO/R, middle cerebral artery occlusion and reperfusion; DHE, dihydroethidium; GSH-Px, glutathione peroxidase; MDA, methane dicarboxylic aldehyde; PTE-5/10, pterostilbene 5 or 10 mg/kg; SOD, superoxide dismutase.

The increase in ROS generation and methane dicarboxylic aldehyde (MDA), superoxide dismutase (SOD) activity, and reduction in glutathione peroxidase (GSH-Px) reflected the level of oxidative stress in the peri-infarct area (31, 32). We used DHE as a marker of ROS in brain tissue sections (Figure 3A) and measured the level of MDA and the activities of SOD and GSH-Px with commercial assay kits, to further evaluate oxidative stress in the peri-infarct area at 24 h after MCAO/R with or without PTE administration. The DHE-positive cell count was significantly higher in the MCAO/R groups (230.7 ± 38.48) than in the Sham group (35.67 ± 27.43). Further, it was also significantly lower in the 10 mg/kg PTE-treated group (145.3 ± 29.7) but not significantly different in the 5 mg/kg PTE-treated group (166.7 + 14.29), compared with the MCAO/R + Vehicle group (Figure 3B, *p* < 0.05).

Compared to the elevated MDA level in the MCAO/R + Vehicle group, the MDA level was lower in the MCAO/R + PTE groups (Figure 3C, *p* < 0.05). The activity of SOD and GSH-Px in the MCAO/R + Vehicle group was significantly higher than in the MCAO/R + PTE groups (Figures 3D,E, *p* < 0.05).

Further pro-inflammatory factors in the peri-infarct area after MCAO/R, PTE administration, or both, were measured by western blot (Figure 3F). At 24 h after the reperfusion (i.e., in the MCAO/R group) the levels of TNF-α, IL-1β, and IL-6 (3.333 ± 0.4136, 1.967 ± 0.1528, 1.667 ± 0.1155) were significantly higher than in the Sham group (Normalized to 1.0). The levels of TNF-α, IL-1β, and IL-6 in 5 mg/kg PTE-treated group had decreased to 2.267 ± 0.3055 (*p* < 0.05), 1.550 ± 0.0500 (*p* < 0.05), 1.433 ± 0.1528 (not significantly). PTE, at 10 mg/kg, significantly reduced the expression of TNF-α, IL-1β, and IL-6 (1.333 ± 0.2082, 1.533 ± 0.1528, 1.533 ± 0.0577) (Figure 3G, *p* < 0.05).

### Effects of PTE on Astrocyte Activation Around the Infarct Region in MCAO/R Mice

**Figure 4:**
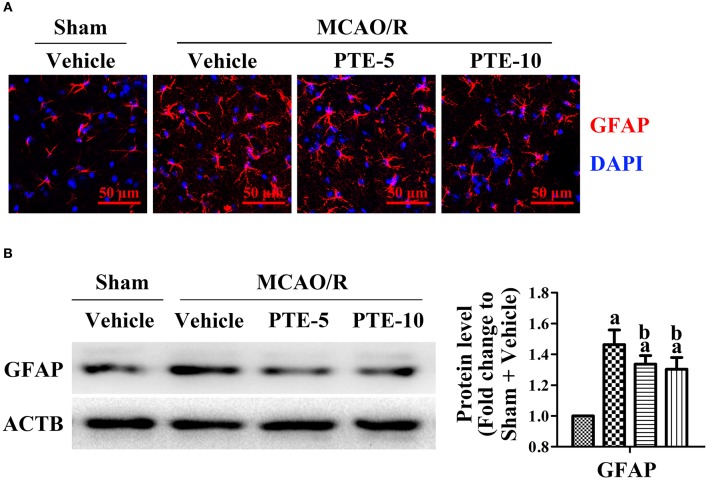
Astrocytes and microglia in the peri-infarct area after MCAO/R with or without PTE administration. **(A)** Activation, proliferation, and aggregation of astrocytes (GFAP, green) were observed by a blinded observer, using immunofluorescence staining on frozen sections fixed at 24 h after MCAO/R or sham surgery. Nuclei were stained with DAPI (blue). Scale bars = 50 μm. **(B)** The protein level of GFAP was analyzed with western blot at 24 h after surgery. ACTB was used as loading control, and the protein level was normalized to Sham + Vehicle group. Values are expressed as mean ± standard deviation (*n* = 6). ^a^*p* < 0.05, compared with Sham + Vehicle. ^b^*p* < 0.05, compared with MCAO/R + Vehicle. Significance was determined using a one-way analysis of variance. ACTB, β-actin; GFAP, glial fibrillary acidic protein; MCAO/R, middle cerebral artery occlusion and reperfusion; PTE-5/10, pterostilbene 5 or 10 mg/kg.

The morphology and quantity of astrocytes around the infarct region were visualized with immunofluorescence staining of GFAP (glial fibrillary acidic protein). We found apparent proliferation, aggregation, and activation of astrocytes in the peri-infarct area (Figure 4A). Further, western blot analysis of the peri-infarct cortex demonstrated increased expression of GFAP, which corroborated with the imaging results (Figure 4B). The number of GFAP-positive astrocytes increased after MCAO/R but were lower in the PTE-treated groups than in the MCAO/R + Vehicle group (Figure 4B, *p* < 0.05).

### Effects of PTE on Expression, Phosphorylation, and Nuclear Translocation of the NF-κB p65 Subunit in the Peri-infarct Area of MCAO/R Mice

**Figure 5:**
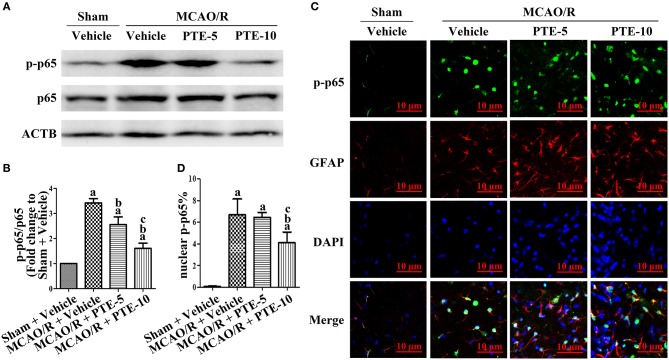
Phosphorylation level of NF-κB p65 subunit in the peri-infarct area after MCAO/R with or without PTE administration. **(A,B)** The protein phosphorylation level of p65 in the peri-infarct cortex was evaluated using western blot analysis at 24 h after MCAO/R or sham surgery. ACTB was used as loading control, and the ratios of p-p65 (S536) to total p65 were normalized to Sham + Vehicle group. **(C)** Expression and distribution of phosphorylated p65 (S536) in astrocytes of the peri-infarct area were observed by a blinded observer, using immunofluorescence double-staining of p-p65 (green) with GFAP (red) and DAPI (blue), respectively, at 24 h after surgery. Scale bars = 10 μm. **(D)** Relative nuclear fluorescence intensity of p-p65 was analyzed by ImageJ. Values are expressed as mean ± standard deviation (*n* = 6). ^a^*p* < 0.05 compared with Sham + Vehicle. ^b^*p* < 0.05, compared with MCAO/R + Vehicle. ^c^*p* < 0.05, compared with MCAO/R + PTE-5. Significance was determined using a one-way analysis of variance. ACTB, β-actin; GFAP, glial fibrillary acidic protein; MCAO/R, middle cerebral artery occlusion and reperfusion; PTE-5/10, pterostilbene 5 or 10 mg/kg.

To unravel the potential role of the transcriptional factor p65 in the MCAO/R brain following PTE administration, total and phosphorylated p65 (phosphorylated at S536) in the peri-infarct area were analyzed using western blotting (Figure 5A). The levels of phosphorylated p65 in the MCAO/R + Vehicle group were significantly higher than in the Sham + Vehicle group. PTE administration remarkably reduced the level of phosphorylated p65 in comparison to the MCAO/R + Vehicle group (Figure 5B, *p* < 0.05). We investigated the distribution of p-p65 (S536) in astrocytes using immunofluorescent double-staining of p-p65 with GFAP and DAPI 24 h after MCAO/R. PTE decreased the level of p-p65 in astrocytes, particularly in the nuclei (Figures 5C,D).

### Effects of PTE on Expression, Phosphorylation, and Nuclear Translocation of the NF-κB p65 Subunit in U251 Astroglioma Cells Subjected to OGD/R

**Figure 6:**
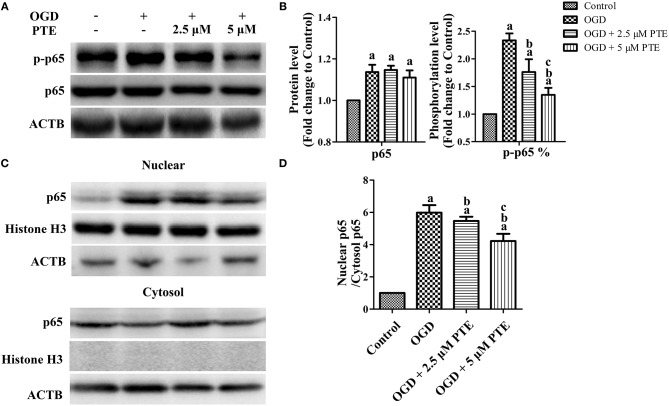
Expression, phosphorylation, and nuclear translocation level of the NF-κB p65 subunit in U251 astroglioma cells subjected to OGD with or without PTE treatment. **(A,B)** Protein and phosphorylation levels of p65 were assessed using western blot analysis at 24 h after OGR in U251 cells subjected to 4 h OGD with or without PTE pre-treatment. ACTB was used as loading control. At the same time **(C,D)**, nuclear translocation of p65 was evaluated using nuclear cytoplasmic extraction followed by western blot analysis. Histone H3 was used as loading control for nuclear proteins and ACTB for cytoplasmic proteins. The expression, phosphorylation (S536), and nuclear translocation level of p65 was normalized to Control. Values are expressed as mean ± SD (*n* = 6). ^a^*p* < 0.05 compared with Control. ^b^*p* < 0.05 compared with OGD. ^c^*p* < 0.05 compared with OGD + 2.5 μM PTE. Significance was determined using a one-way analysis of variance. ACTB, β-actin; OGR, oxygen-glucose reintroduction; OGD, oxygen-glucose deprivation; PTE, pterostilbene.

**Figure 7:**
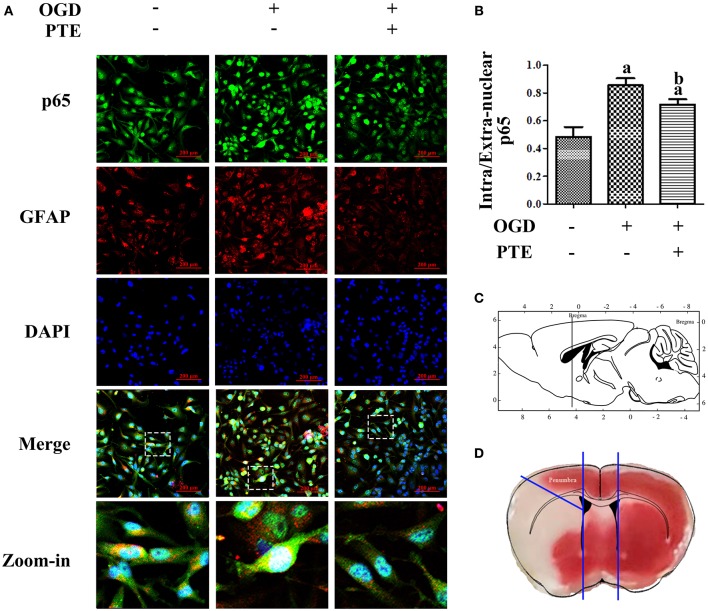
Intracellular distribution of p65 in U251 astroglioma cells subjected to OGD/R with or without PTE pre-treatment. **(A,B)** OGD/R-induced nuclear translocation of p65 was further observed by an observer blinded to the groups, using immunofluorescence double-staining of p65 (green) and GFAP (red) under the intervention of PTE (5 μM). Nuclei were re-stained with DAPI (blue), and scale bars = 200 μm. **(C,D)** Schematic diagram of the location of the penumbra where the representative vision was acquired. The sketch in **(C)** is referenced from *Page 77, The Mouse Brain in Stereotaxic Coordinates (second edition), 2001, ACADEMIC PRESS*. GFAP, glial fibrillary acidic protein; OGD/R, oxygen-glucose deprivation and reintroduction; PTE, pterostilbene.

To investigate the change in p65 in astrocytes following ischemia-reperfusion, we employed an oxygen-glucose deprivation and reperfusion (OGD/R) model in U251 astroglioma cells to simulate MCAO/R *in vitro*. The expressions of p65 after OGD/R, with or without PTE pre-treatment, were analyzed with western blot and nuclear-cytoplasmic extraction. PTE (2.5 or 5 μM) had no effect on p65 expression in the U251 cells subjected to OGD/R. However, the levels of phosphorylated p65 (S536) remarkably declined in PTE pre-treated groups (Figures 6A,B, *p* < 0.05). The nuclear translocation levels of p65 in the PTE-treated groups were lower than in the OGD group (Figures 6C,D, *p* < 0.05). Moreover, immunofluorescence staining of p65, GFAP, and DAPI demonstrated that 5 μM PTE remarkably decreased the nuclear translocation of p65 that was induced by OGD (Figures 7A,B).

### Effects of PTE on Inflammation, Oxidative Stress, and Neuron Death in HT22 Hippocampal Cells Co-cultured With U251 Astroglioma Cells Subjected to OGD/R

**Figure 8:**
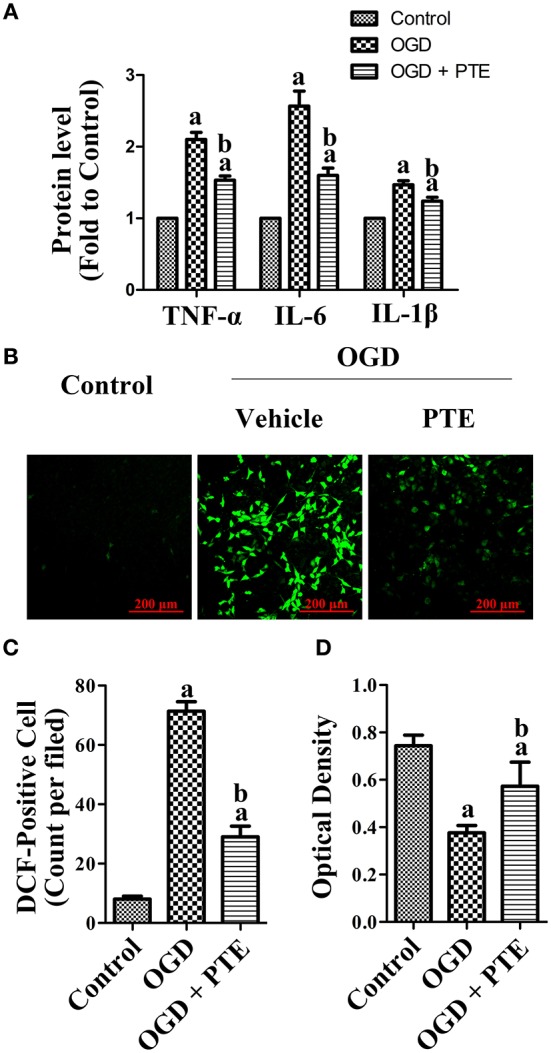
Pro-inflammatory factor generation in culture media of U251 astroglioma cells subjected to OGD/R, and oxidative stress-induced neural death in HT22 cells co-cultured with U251 pre-treated with OGD/R. **(A)** Levels of TNF-α, IL-6, and IL-1β in culture media were evaluated using ELISA kits at 24 h after OGD/R for each group. The results were normalized to Control. **(B)** ROS-generation in HT22 hippocampal cells was observed by a blinded observer, using DCF staining. **(C)** DCF-positive cells were counted manually and calculated as an average of five random visual fields for each dish. **(D)** Cell viability of HT22 was assessed using CCK-8 assay after co-culturing with OGD-pre-treated U251 for 24 h. The concentration of PTE is 5 μM. Values are expressed as mean ± SD (*n* = 6). ^a^*p* < 0.05, compared with Control. ^b^*p* < 0.05, compared with OGD. Significance was determined using a one-way analysis of variance. CCK-8, cell counting kit-8; DCF, 2′-7′-dichlorofluorescein diacetate; ELISA, enzyme-linked immunosorbent assay; IL, interleukin; OGR, oxygen-glucose reintroduction; OGD/R, oxygen-glucose deprivation and reintroduction; PTE, pterostilbene; ROS, reactive oxygen species; TNF, tumor necrosis factor.

The levels of TNF-α, IL-1β, and IL-6 that were detected in the U251 culture media with enzyme-linked immune-sorbent assays (ELISAs) were increased after OGD/R. Further, the levels of TNF-α, IL-1β, and IL-6 were decreased in the OGD + PTE (5 μM) group compared with in the OGD group (Figure 8A, *p* < 0.05). The oxidative stress and viability of HT22 cells that were co-cultured with U251 cells via a transwell insert were detected using 2′-7′-dichlorofluorescein diacetate (DCF) staining and a Cell Counting Kit-8 (CCK-8), respectively. PTE pre-treatment significantly decreased DCF-positive cell counts 24 h after OGD/R compared with vehicle (Figures 8B,C, *p* < 0.05). Cell viability, however, was remarkably higher in the OGD + PTE group in comparison to the OGD group (Figure 8D, *p* < 0.05).

## Discussion

In the present study, we elucidated the effect of PTE on attenuating inflammation and oxidative injury after focal cerebral IR in mice. *In vivo*, PTE played a neuroprotective role by decreasing infarct volume, reducing brain edema, and improving neurological scores following transient focal cerebral IR. It also significantly reduced the neuronal apoptosis around the infarct zone and improved the survival rate of neuronal cells *in vitro*. However, the impact of PTE on the long-term survival rate of mice was not significant. These results may indicate that PTE is more meaningful in improving the prognosis and quality of survival than in prolonging life.

AIS, accounting for about 6.5 million deaths every year and forecasted to result in the annual loss of over 200 million disability-adjusted life years by 2030 (33), had been one of the major causes of morbidity and mortality worldwide (1).

The prognosis of AIS is still unsatisfactory, although great breakthroughs have been made in the research and development of diagnosis technology, neuroimaging, medical reperfusion therapy, and surgical reperfusion therapy (34). Besides, adjuvant medications aiming to reduce permanent brain injury and neurological function impairment are also under active investigation (35).

Current neuroprotective drugs had shown little benefit for consequential injury after AIS (2). However, PTE, the naturally occurring 3′, 5′-dimethylated analog of resveratrol, had been indicated to show broad anti-inflammatory and anti-oxidative stress ability (12, 36); these are both among the neuroprotective strategies after cerebral ischemia (33).

Emerging evidence suggests that the inflammatory signal plays a pivotal role in secondary injury following IR (3). It has been shown to be involved in all stages of the cerebral ischemic-reperfusion injury process from arterial occlusion to regenerative processes during neurorepair (37) and has been considered one of the prime targets for the development of new stroke therapies (38). Both innate and adaptive immunity are involved in this process. More importantly, the modulation of adaptive immunity has been found to exert a remarkable protective effect on AIS (37).

PTE has previously been shown to attenuate lipopolysaccharide-induced learning and memory impairments by microglia inhibition and neuronal protection (39). It was also suggested in our previous study that PTE can attenuate early brain injury following subarachnoid hemorrhage, possibly via the inhibition of NLRP3 inflammasome and Nox2-related oxidative stress (12).

The pathological role of oxidative stress had been demonstrated in a variety of central nervous system diseases, including neurodegenerative, ischemic, infectious, and traumatic disorders. The brain is more vulnerable to oxidative damage due to its high metabolic demand (40). In the brain, oxidative stress is closely related to inflammation and vice versa (41). Oxidative status, as a result of inflammation, also plays a crucial role in activating inflammation (42), which subsequently causes neuronal apoptosis (43). Overall, inflammation and oxidative stress are undoubtedly valid intervention targets for IR injury after cerebral stroke (2, 11, 12, 44).

It has been demonstrated that PTE can attenuate global cerebral ischemia-reperfusion injury by inducing mitochondrial oxidative injury (11). Additionally, at a diet-achievable dose, PTE acted as a potent neuromodulator in aging and Alzheimer's disease, probably driven by increased peroxisome proliferator-activated receptor α expression (13).

In ischemic tissue, ATPase-dependent biological processes are disrupted, which, in turn, induces intracellular calcium overload and lysis of organelle and plasma membranes (45). Even worse, although, following reperfusion, tissue has been shown to recover from an oxygen-starved status and generate excessive ROS, triggering peroxidation and inflammatory responses and the generation of MDA, the end-product of lipid oxidation (45, 46). In this process, anti-oxidative enzymes, including GSH-Px and SOD, which scavenged excess ROS and reduced its toxic effects, were exhausted (45).

In the current study, the data showed that PTE remarkably reduced the levels of oxidative stress and inflammation in the peri-infarct brain region after reperfusion and in co-cultured neuronal cells, as evidenced by the decreased level of ROS, MDA, and pro-inflammatory factors TNF-α, IL-1β, and IL-6 and the increased activity of SOD and GSH-Px.

A considerable number of pathology processes, including glial cell activation, peripheral immune cell infiltration, capillary pericyte constriction, et al., have been shown to be involved in inflammation-related post-stroke secondary injury (38). Astrocytes, the most abundant type of glial cell in the brain, were closely associated with this process (5).

The activation of astrocytes, presenting as astrocyte proliferation, morphological change, and enhanced expression of GFAP, can be induced by cerebral ischemia, inflammation, and oxidative stress. The post-stroke glial scar, as a result of astrocyte activation and proliferation, has been shown to form a dense barrier against neuroregeneration (5). Activated astrocytes also generated TNF-α, IL-1β, IL-6, and cyclooxygenase-2, which in turn further promoted the activation of astrocytes (47, 48).

Moreover, astrocytes play an important role in physiological conditions. Dysregulation of normal astrocyte physiology leads to post-stroke impairments. For example, the network of communication channels throughout the brain constructed by astrocyte syncytium would be impaired after AIS. It would also lead to the disfunction of cytokine production, ion homeostasis, and blood flow regulation (33).

Previous studies successively revealing the post-stroke role of astrocytes remained insufficient owing to the diversity of astrocyte functions. New evidence was still needed. In the present study, the proliferation of astrocytes around the infarct zone was observed. GFAP, an astrocytic marker, was also significantly increased in the peri-infarct area, which was abolished by the PTE treatment. This evidence may indicate an important role for astrocytes in the progress of IRI and in eliciting the protective effect of PTE.

It was previously demonstrated that NF-κB pathway regulation might be one of the anti-inflammation mechanisms of PTE (49–51). However, to the best of our knowledge, no evidence about the NF-κB signal-regulating effect of PTE on astrocytes and cerebrovascular disease had been reported previously.

The activation and functional status of the NF-κB pathway have been found to be regulated by a complicated upstream signaling pathway together with a wide range of regulatory factors (52–54). It can be mediated by the canonical pathway, involving TLRs, proinflammatory cytokines, IKKβ, IKKγ, and IκB et al., or the non-canonical pathway, involving LTβ, CD40L, BAFF, RANKL, IKKα, and p100 et al. (55, 56).

The NF-κB p65 subunit, activated by the canonical pathway, is a transcription factor that can be activated by hypoxia, ROS, and several inflammatory mediators. The phosphorylation and nuclear translocation of p65 is one of the most direct and appropriate indicators for evaluating the activation and functional status of the NF-κB pathway (57). It is involved in astrocyte activation, generation of pro-inflammatory factors IL-6, IL-1β, and TNF-α, and macrophage and T-cell infiltration, thus leading to secondary inflammatory injury in central nervous system diseases (15–19).

In our study, PTE decreased the phosphorylation of p65 (S536) induced by IR. The increased expression, phosphorylation, and nuclear translocation of p65 in U251 astrocytoma cells, following OGD/R, were reduced by PTE treatment. In brief, PTE inhibited NF-κB in astrocytes after stroke and presented an anti-inflammatory effect.

Unfortunately, the upstream regulatory mechanism of p65 phosphorylation and nuclear translocation remain unexplained in the present study. The polyubiquitination and subsequent degradation of IκB might be one of the most direct regulating factors (58, 59). However, the whole upstream signal pathway should be considered deliberately if we want to reveal the underlying mechanism of the regulating effect of PTE on NF-κB activation. This is one of the directions that should be focused upon in the future.

In our previous study, PTE showed protective effects against mitochondrial oxidative stress and glutamate-induced neuronal oxidative injury (11, 28). It must be noted that, in the *in vivo* experiment of the current study, PTE might not only have affected astrocytes but also have had an effect on neurons as well as other cell types. The anti-oxidative property of PTE is likely to have improved the tolerance of neurons to ischemia, mediating its effect against inflammatory injury. However, this theory also requires further validation.

Besides, we did not focus on the effect of PTE on microglia activation in the present study, though this has been found to play an essential role in inflammatory responses and should not be ignored (60). Depending on the activated phenotype, microglia have biphasic functions in ischemic stroke (61). M1 microglia exacerbates neuronal injury by secreting pro-inflammatory cytokines, while M2 microglia is a neuroprotective mediator (62). Under certain conditions, microglia can restrict ischemia-induced astrocyte response and provide neuroprotective effects (63). However, an increasing number of studies have reported that over-activated microglia mediate inflammatory injury after stroke. The pro-inflammatory cytokines secreted by microglia can be able to amplify the inflammatory activation of astrocytes (64). Thus, the inhibition of microglia activation might be therapeutic for AIS (47, 65, 66). The effects of PTE on microglial activation after AIS and the subsequent pro-inflammatory cytokine releases should also be worked on in the future.

In conclusion, our findings in mice and in HT22/U251 cell co-cultures suggested that PTE treatment presents a promising cerebral-protective effect against IRI, involving inhibition of astrocyte-mediated inflammation and associated oxidative stress. This effect may partially result from the inhibited phosphorylation and nuclear translocation of NF-κB. The therapeutic application of PTE in AIS, involving several intervention targets, may be a promising strategy for stroke management.

## Data Availability Statement

All datasets generated for this study are included in the manuscript/Supplementary Files.

## Ethics Statement

This study was carried out in accordance with the recommendations of Association for the Assessment and Accreditation of Laboratory Animal Care International (AAALAC) standards and Ethics Committee of the Fourth Military Medical University. The protocol was approved by the Ethics Committee of the Fourth Military Medical University.

## Author Contributions

YQ conducted this project and supported the research. BW and HL designed the study and drafted the manuscript. JL, XWa, HB, LY, and XLi performed the experiments and acquired the primary data. XWu, HG, MS, and XLiu completed the statistics and interpreted the data. DF, LZ, and WG revised the manuscript. All authors reviewed and approved the final.

### Conflict of Interest

The authors declare that the research was conducted in the absence of any commercial or financial relationships that could be construed as a potential conflict of interest.
